# Supplementary material for: Development of a Recombinant Single-Cycle Influenza Viral Vector as an Intranasal Vaccine against SARS-CoV-2 and SARS-like Betacoronaviruses
Source: bioRxiv. 2025 Dec 1:2025.11.28.691177. Preprint. [Version 1] doi: 10.1101/2025.11.28.691177 (PMC12694603; doi:10.1101/2025.11.28.691177)
Supplement: 1 [file NIHPP2025.11.28.691177V1-supplement-1.pdf]

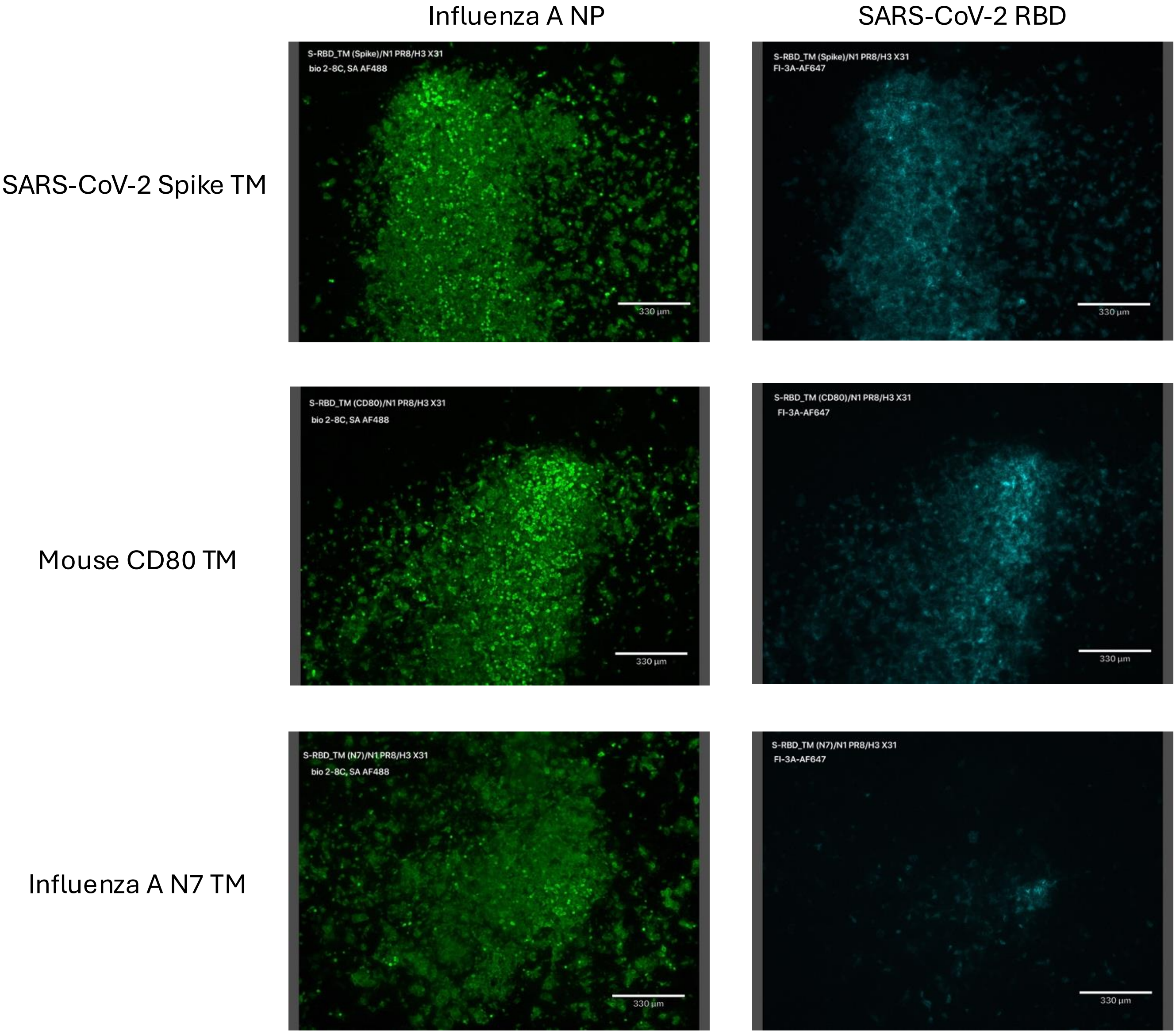

Figure S1: Expression of influenza NP and RBD in cell culture infected with S-RBD-TM with various TM. MDCK cells seeded in 6-well plate were infected with S-RBD-TM (fused to a SARS-CoV-2 Spike TM, mouse CD80 TM or an influenza neuraminidase N7 transmembrane domain). 16 hours post infection, cells were formalin fixed, permeabilised with 0.5% Triton-X 100 and stained with an RBD-specific IgG1 (FI-3A) labelled with AF647, and a biotinylated anti-Influenza NP IgG1 (2-8C biotin), followed by Streptavidin labelled with AF488. Stained cells were visualised using a fluorescence microscope. Scale bar: 330uM. (TM: transmembrane domain, AF647: Alexa Fluor 647, AF488: Alexa Fluor 488)

SARS-CoV-2 Variant	G339	R346	S371	S373	S375	K417	N440	G446	L452	N460	S477	T478	E484	F486	Q493	G496	Q498	N501	Y505
Wuhan	.	.	.	.	.	.	.	.	.	.	.	.	.	.	.	.	.	.	.
Alpha (B.1.1.7)	.	.	.	.	.	.	.	.	.	.	.	.	.	.	.	.	.	N501Y	.
Beta (B.1.351)	.	.	.	.	.	K417N	.	.	.	.	.	.	E484K	.	.	.	.	N501Y	.
Gamma (P.1)	.	.	.	.	.	K417T	.	.	.	.	.	.	E484K	.	.	.	.	N501Y	.
Delta (B.1.617.2)	.	.	.	.	.	.	.	.	L452R	.	.	T478K	.	.	.	.	.	.	.
Omicron BA.1	G339D	.	S371L	S373P	S375F	K417N	N440K	G446S	.	.	S477N	T478K	E484A	.	Q493R	G496S	Q498R	N501Y	Y505H
Omicron BA.2	G339D	.	.	.	.	K417N	N440K	.	.	.	S477N	T478K	E484A	.	Q493R	.	Q498R	N501Y	.
Omicron BA.4 / BA.5	G339D	.	.	.	.	K417N	N440K	.	L452R	.	S477N	T478K	E484A	F486V	Q493R	.	Q498R	N501Y	.
BA.2.75	G339D	.	.	.	.	.	N440K	G446S	.	.	S477N	T478K	E484A	.	Q493R	.	Q498R	N501Y	.
XBB / XBB.1.5	G339D	R346T	.	.	.	.	.	G446S	.	N460K	.	.	.	.	.	.	.	.	F486P / F486S

Table S1: Position-by-position mutation matrix of the SARS-CoV-2 spike RBD (residues 319–541). The table shows the specific amino acid changes in major variants relative to Wuhan SARS-CoV-2. Each column represents an RBD residue and each row a variant. Cells list the observed amino acid substitution at each position; blank cells indicate identity with the reference sequence.
